# Supplementary material for: Intestinal lysozyme1 deficiency alters microbiota composition and impacts host metabolism through the emergence of NAD+-secreting ASTB Qing110 bacteria
Source: mSystems. 2024 Feb 16;9(3):e01214-23. doi: 10.1128/msystems.01214-23 (PMC10949482; doi:10.1128/msystems.01214-23)
Supplement: Supplemental Material — Supplemental figures. [file msystems.01214-23-s0001.docx]

Supplementary Figure 1


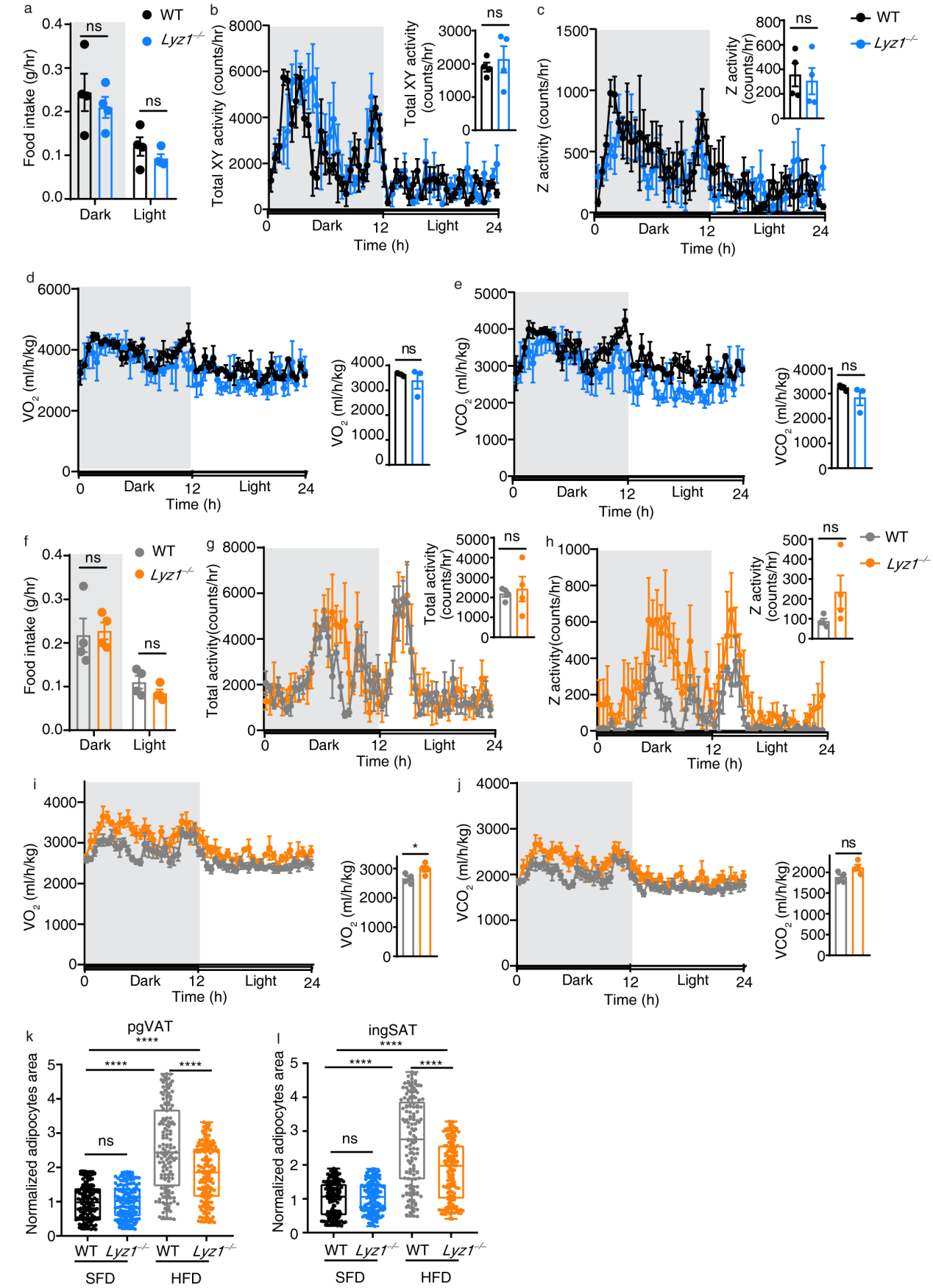


**Supplementary figure 1. Metabolic Profiles of Separately Housed WT and *Lyz1^−/−^* Mice on Different Diets.**

(a) Hourly food intake of 10-week-old WT and *Lyz1^−/−^* mice on a standard chow diet (SFD).

(b) Total XY activity of 10-week-old mice plotted over a 24-hour period.

(c) Z activity of 10-week-old mice plotted over a 24-hour period.

(d) Oxygen consumption (VO_2_) of 10-week-old mice plotted over a 24-hour period.

(e) Carbon dioxide production (VCO_2_) of 10-week-old mice plotted over a 24-hour period.

(f) Hourly food intake of 19-week-old WT and *Lyz1^−/−^* mice on a high-fat diet (HFD).

(g) Total XY activity of 19-week-old mice plotted over a 24-hour period.

(h) Z activity of 19-week-old mice plotted over a 24-hour period.

(i) Oxygen consumption (VO_2_) of HFD-fed 19-week-old mice plotted over a 24-hour period.

(j) Carbon dioxide production (VCO_2_) of HFD-fed 19-week-old mice plotted over a 24-hour period.

(k) Quantification of adipocyte area in perigonadal visceral adipose tissue (pgVAT) of WT and *Lyz1^−/−^* mice on SFD or HFD.

(l) Quantification of adipocyte area in inguinal subcutaneous adipose tissue (ingSAT) of WT and *Lyz1^−/−^* mice on SFD or HFD.

Individual data points in panels (a-j) are represented by symbols, with means (±SEM) displayed. Mean values with error bars indicating SEM are shown in panels (k, l). Statistical analysis employed multiple t tests in (a, f), two-tailed Student’s t tests in (b-e, g-j), or one-way ANOVA with Tukey's multiple comparisons test in (k, l). "NS" indicates no significant difference (P>0.05), while asterisks denote significance levels: *P<0.05, ****P<0.0001. The data presented is representative of results from at least three independent experiments.

Supplementary Figure 2

**
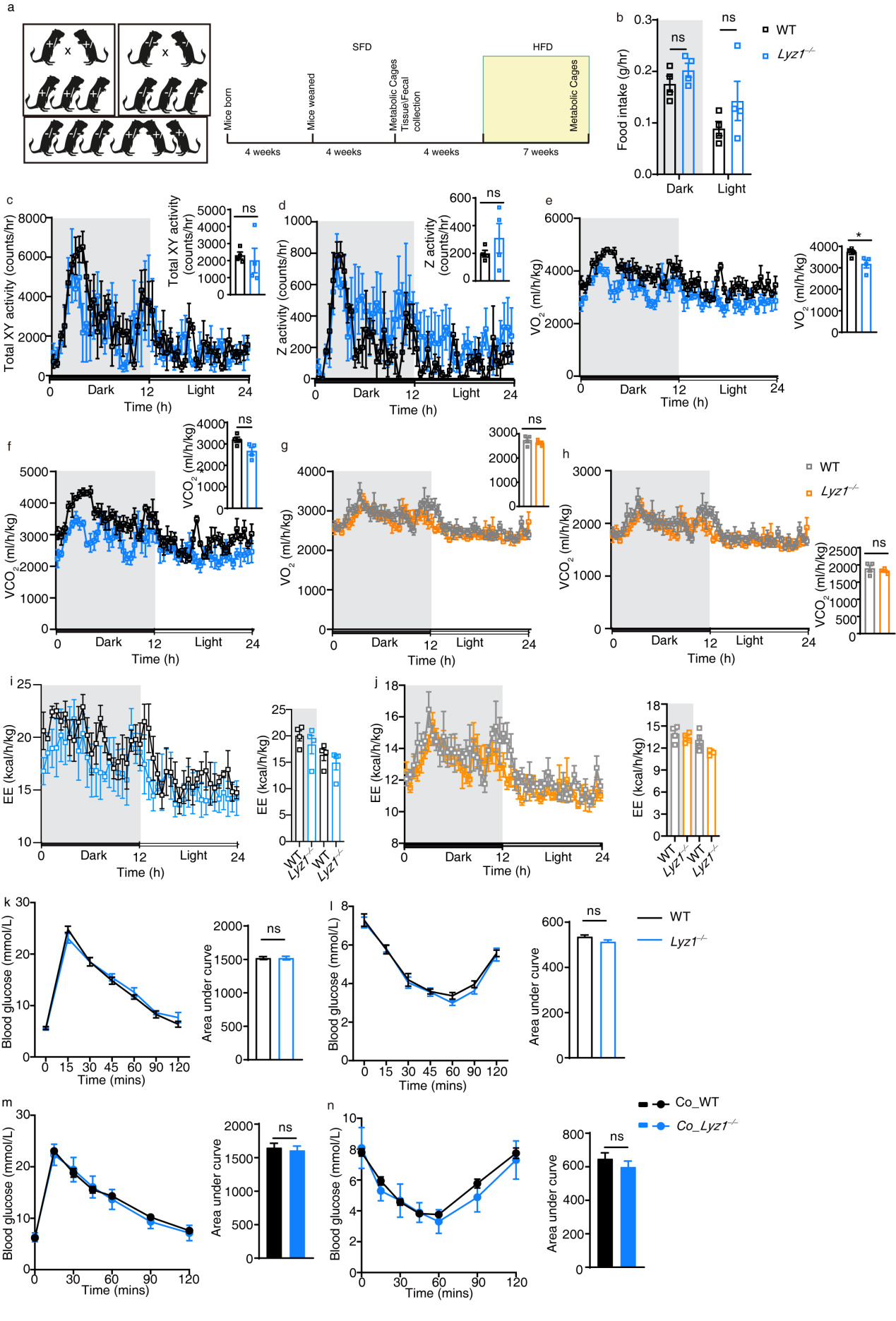
Supplementary figure 2. Metabolic profiles of cohoused WT and *Lyz1^−/−^* mice.**

(a) Schematic representation of the feeding scheme.

(b) Hourly food intake of cohoused 10-week-old WT and *Lyz1^−/−^* mice on SFD.

(c) Total XY activity of cohoused 10-week-old WT and Lyz1^−/−^ mice on SFD plotted against time over a 24-hour period.

(d) Z activity of cohoused 10-week-old WT and Lyz1^−/−^ mice on SFD plotted against time over a 24-hour period.

(e) Oxygen consumption (VO_2_) of cohoused 10-week-old WT and *Lyz1^−/−^* mice plotted over a 24-hour period.

(f) Carbon dioxide production (VCO_2_) of cohoused 10-week-old WT and *Lyz1^−/−^* mice on SFD plotted against time over a 24-hour period.

(g) Oxygen consumption (VO_2_) of cohoused 19-week-old WT and *Lyz1^−/−^* mice on HFD plotted against time over a 24-hour period.

(h) Carbon dioxide production (VCO_2_) of cohoused 19-week-old WT and *Lyz1^−/−^* mice on HFD plotted against time over a 24-hour period.

(i) Energy expenditure (EE) of cohoused 10-week-old WT and Lyz1^−/−^ mice on SFD plotted against time over the course of one day period.

(j) Energy expenditure (EE) of cohoused 19-week-old WT and *Lyz1^−/−^* mice on HFD plotted against time over the course of one day period.

(k) Plasma glucose concentration and mean area under the curve measured during an intraperitoneal glucose tolerance test (ipGTT) in separated-housed WT and Lyz1^−/−^ mice on SFD (n=8 mice per group).

(l) Plasma glucose concentration and mean area under the curve measured during an insulin tolerance test (ITT) in separated-housed WT and Lyz1^−/−^ mice on SFD (n=8 mice per group).

(m) Plasma glucose concentration and mean area under the curve measured during an intraperitoneal glucose tolerance test (ipGTT) in cohoused WT and Lyz1^−/−^ mice on SFD (n=14 mice per group).

(n) Plasma glucose concentration and mean area under the curve measured during an insulin tolerance test (ITT) in cohoused WT and Lyz1^−/−^ mice on SFD (n=14 mice per group).

Means (±SEM) are plotted with each symbol representing an individual animal in (b-j). Mean values are plotted with bars indicating SEM in (k-n). P values were calculated with multiple t tests in (b), two-tailed Student’s t tests in (c-h, k-n), or one-way ANOVA with Tukey's multiple comparisons test in (i, j). NS indicates no significant difference (P>0.05). *P<0.05. Data are representative of at least three independent experiments.


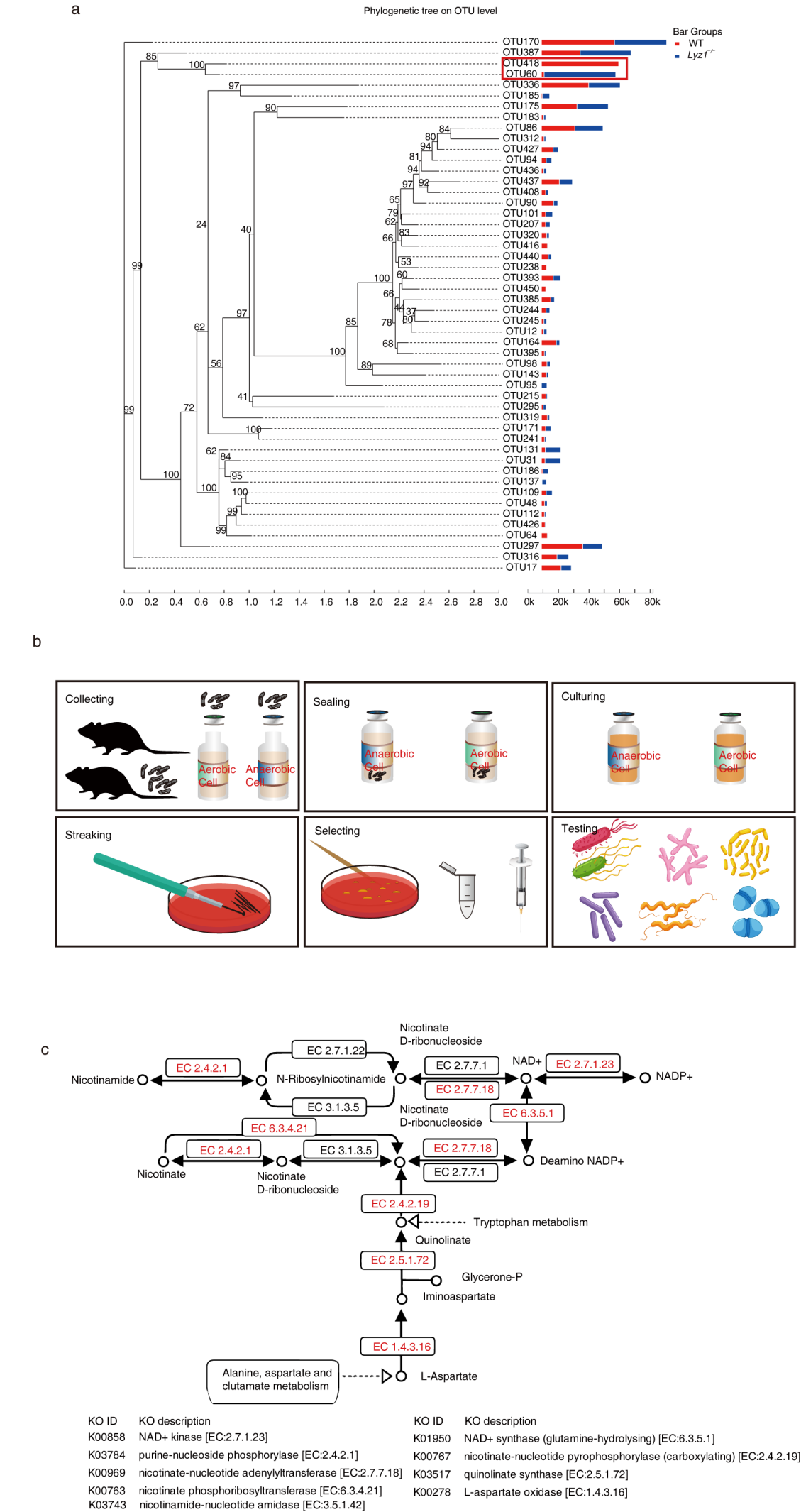


Supplementary Figure 3

**Supplementary Figure 3. Bacterial Isolation and Genomic Analysis.**

1. Phylogenetic tree depicting the top 50 abundant OTUs found in mice fecal samples. OTU 418 and OTU 60 are closely related in evolution.

(d) Schematic illustration outlining the steps involved in bacterial isolation and characterization.

(e) Diagram representing NAD^+^ biosynthesis pathways in bacteria. NAD^+^ can be synthesized through de novo pathways or salvaged from intermediates and small precursors like NA (nicotinic acid), NAM (nicotinamide), and NR (nicotinamide riboside). Our study indicates that *ASTB Qing110* possesses complete NAD^+^ biosynthesis pathways, with essential enzymatic genes highlighted in red.

Supplementary Figure 4


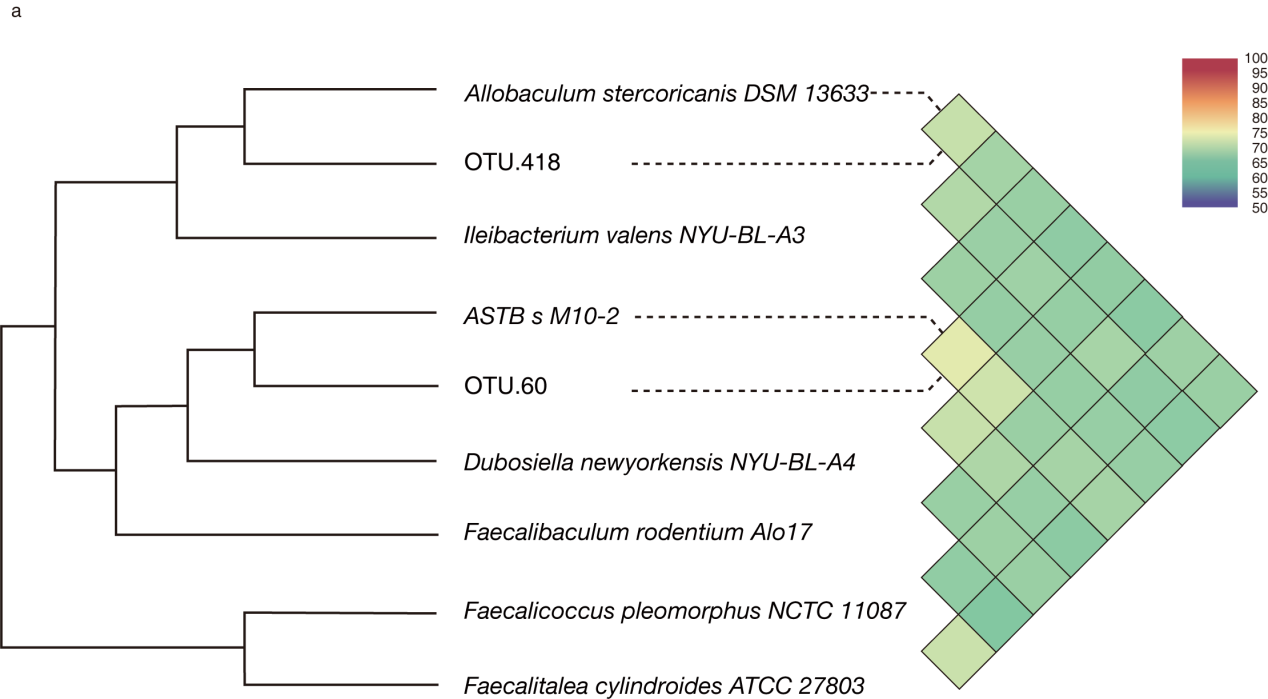


**Supplementary Figure 4. Whole-Genome Analysis of ASTB Qing110 and Related Bacteria.**

(a) Average Nucleotide Identity (ANI) comparison of the genomes of *Qing110* and those of bacteria taxonomically related to *Qing110.*

Supplementary Figure 5


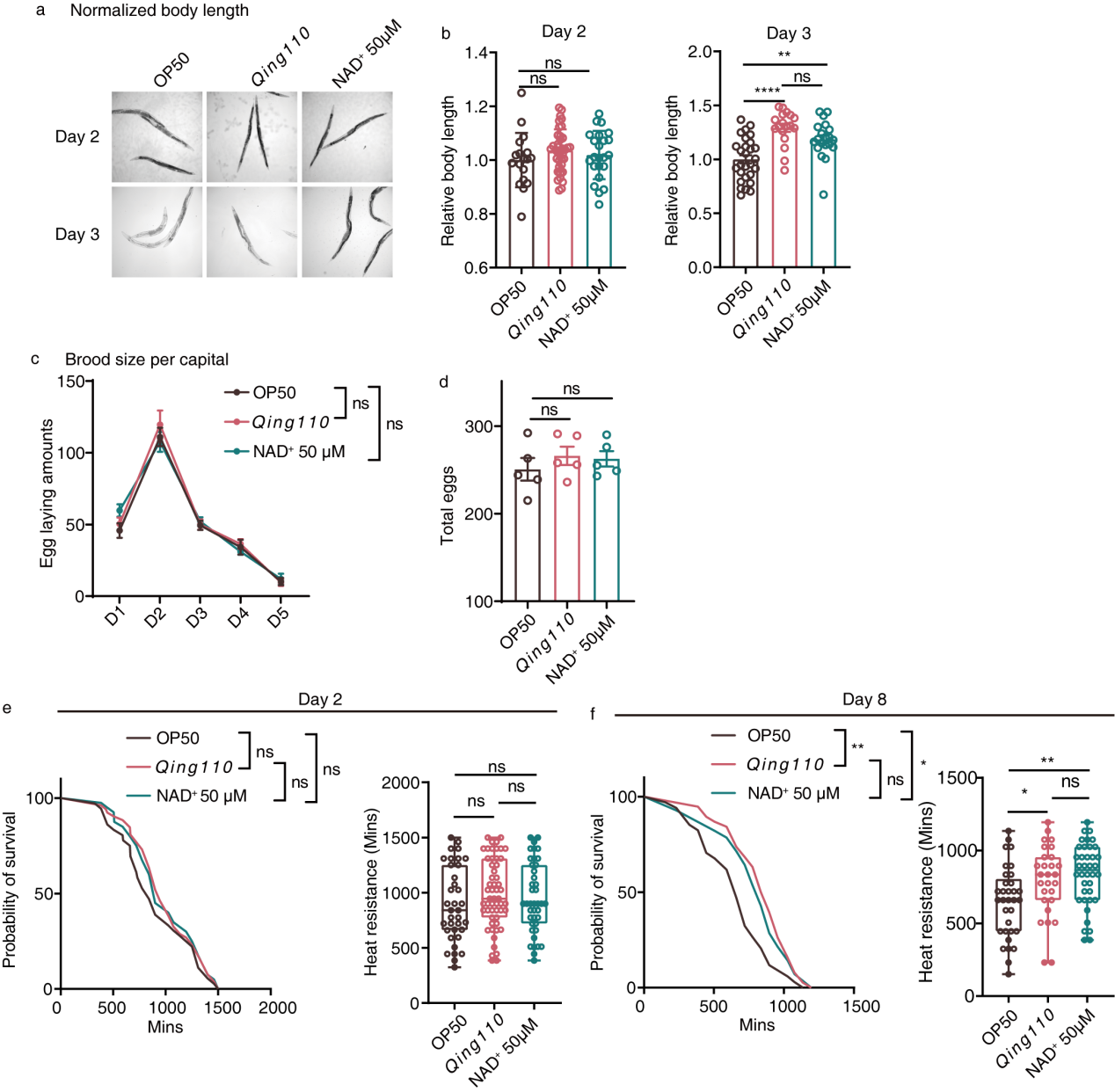


**Supplementary Figure 5. *Qing110* Supplementation Enhances Healthspan in *C. elegans*.**

(a) Images of day 2 and day 3 adult N2 worms treated with *E. coli* OP50, *Qing110* and NAD^+^ (50 µM).

(b) Measurement of the relative body length of N2 worms.

(c) Brood size per capital of N2 worms.

(d) Total eggs laid by N2 worms.

(e) Survival curves and mean survival time of day 2 adult N2 worms under heat stress (37 °C).

(f) Survival curves and mean survival time of day 8 adult N2 worms under heat stress (37 °C).

Mean values are presented with error bars indicating SEM in panel (c). Means (±SEM) are plotted with each symbol representing an individual animal in panels (b, d-f). Statistical analysis was conducted using one-way ANOVA with Tukey's multiple comparisons test in panels (b, d-f) or Log-rank (Mantel-Cox) test in panels (c, e, f). "NS" indicates no significant difference (P>0.05), while asterisks denote significance levels: *P<0.05, **P<0.01, ****P<0.0001.

Supplementary Figure 6


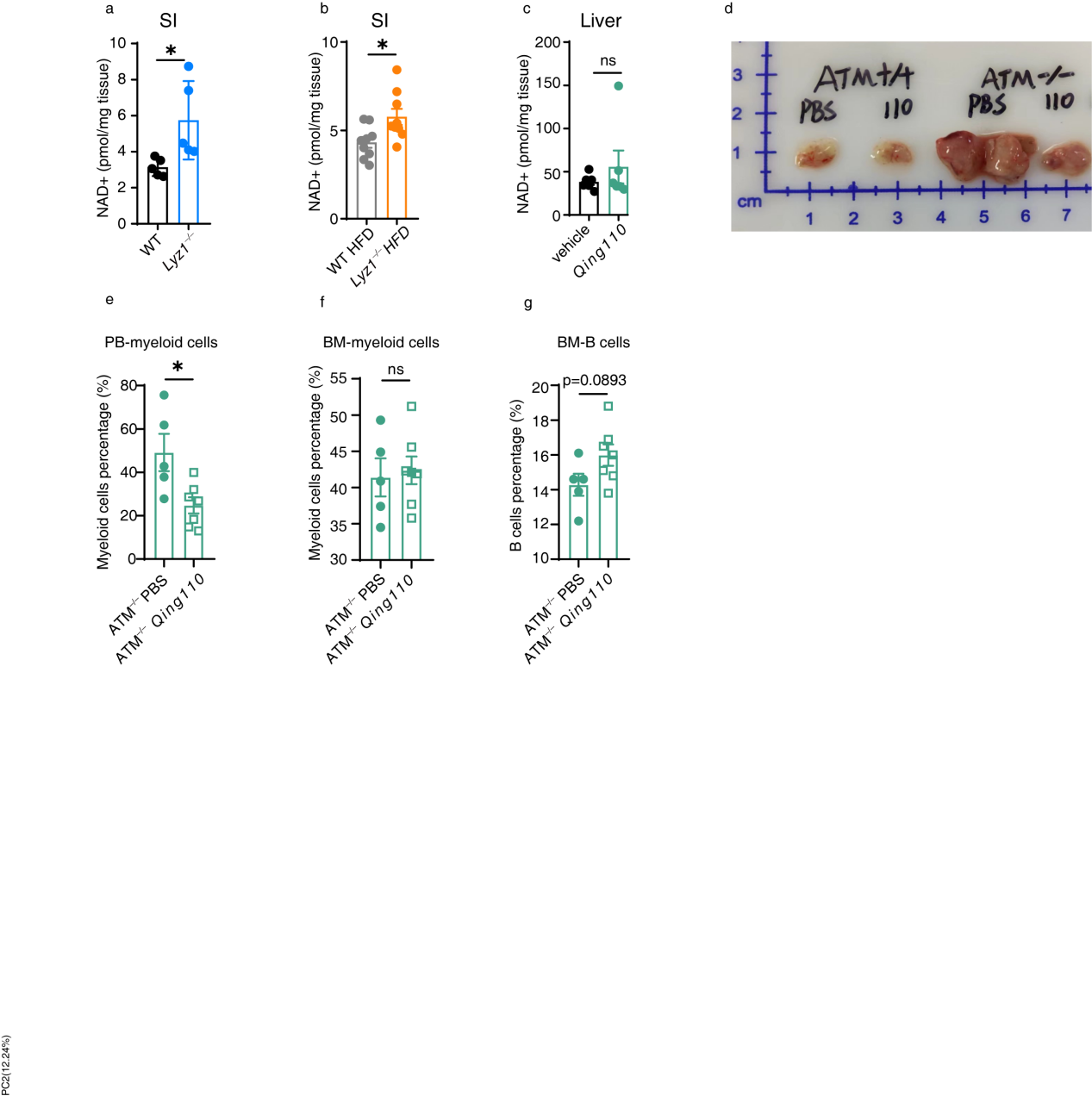


**Supplementary figure 6. Effects of Qing110 Treatment on Various Parameters.**

1. Measurement of NAD^+^ levels in small intestinal epithelial cells of WT and *Lyz1^−/−^* mice.
2. Measurement of NAD^+^ levels in small intestinal epithelial cells of WT and *Lyz1^−/−^* mice after HFD treatment after 7 weeks of high-fat diet feeding.

(c) Measurement of NAD^+^ levels in the liver after 1-month treatment of PBS or *Qing110* via oral gavage.

(d) The representative images of thymus from 100-day-old WT and *Atm^−/−^* mice treated with PBS or *Qing110*.

(e) Abundance of myeloid cells in peripheral blood examined by flow cytometry and compared between PBS-treated and *Qing110*-treated *Atm^−/−^* mice.

(f) Abundance of myeloid cells in bone marrow by flow cytometry and compared between PBS-treated and *Qing110*-treated *Atm^−/−^* mice.

(g) Abundance of B cells in bone marrow by flow cytometry and compared between PBS-treated and *Qing110*-treated *Atm^−/−^* mice.

Means (±SEM) are plotted with each symbol representing an individual animal in panels (a, b, c, f, g, h). Statistical analysis was conducted using two-tailed Student’s t tests in panels (a, b, c, f, g, h). "NS" indicates no significant difference (P>0.05), while asterisks denote significance levels: *P<0.05.
